# Mining transcriptomic data to identify *Saccharomyces cerevisiae* signatures related to improved and repressed ethanol production under fermentation

**DOI:** 10.1371/journal.pone.0259476

**Published:** 2022-07-26

**Authors:** Sima Sazegari, Ali Niazi, Zahra Zinati, Mohammad Hadi Eskandari

**Affiliations:** 1 Institute of Biotechnology, Shiraz University, Shiraz, Fars, Iran; 2 Department of Agroecology, College of Agriculture and Natural Resources of Darab, Shiraz University, Shiraz, Fars, Iran; 3 Department of Food Science and Technology, Shiraz University, Shiraz, Fars, Iran; Osmania University, INDIA

## Abstract

*Saccharomyces cerevisiae* is known for its outstanding ability to produce ethanol in industry. Underlying the dynamics of gene expression in *S*. *cerevisiae* in response to fermentation could provide informative results, required for the establishment of any ethanol production improvement program. Thus, representing a new approach, this study was conducted to identify the discriminative genes between improved and repressed ethanol production as well as clarifying the molecular responses to this process through mining the transcriptomic data. The significant differential expression probe sets were extracted from available microarray datasets related to yeast fermentation performance. To identify the most effective probe sets contributing to discriminate ethanol content, 11 machine learning algorithms from RapidMiner were employed. Further analysis including pathway enrichment and regulatory analysis were performed on discriminative probe sets. Besides, the decision tree models were constructed, the performance of each model was evaluated and the roots were identified. Based on the results, 171 probe sets were identified by at least 5 attribute weighting algorithms (AWAs) and 17 roots were recognized with 100% performance Some of the top ranked presets were found to be involved in carbohydrate metabolism, oxidative phosphorylation, and ethanol fermentation. Principal component analysis (PCA) and heatmap clustering validated the top-ranked selective probe sets. In addition, the top-ranked genes were validated based on GSE78759 and GSE5185 dataset. From all discriminative probe sets, *OLI1* and *CYC3* were identified as the roots with the best performance, demonstrated by the most weighting algorithms and linked to top two significant enriched pathways including porphyrin biosynthesis and oxidative phosphorylation. *ADH5* and *PDA1* were also recognized as differential top-ranked genes that contribute to ethanol production. According to the regulatory clustering analysis, *Tup1* has a significant effect on the top-ranked target genes *CYC3* and *ADH5* genes. This study provides a basic understanding of the *S*. *cerevisiae* cell molecular mechanism and responses to two different medium conditions (Mg^2+^ and Cu^2+^) during the fermentation process.

## Introduction

*Saccharomyces cerevisiae* is used as one of the main microorganisms for bio-ethanol production in research and industry. In addition to high ethanol production potential, stability for anaerobic fermentation and low pH tolerance, facilitates its use in industry for ethanol production [1]. Among industrial *S*. *cerevisiae strains used in fermentation industry*, *JP1* is one of the dominants exhibits high fermentation rate due to high temperature tolerance and low pH stability [2]. Since many of *S*. *cerevisiae* genes have been functionally annotated and characterized, genetic manipulation of this organism is well developed [3–5]. Several research have been conducted on the *S*. *cerevisiae* metabolic engineering to generate efficient ethanol producing strains [6, 7]. Suji et al [8], for example used the *PHO13* deletion in conjunction with *LAD1* and *ALX1* heterologous expression to improve *S*. *cerevisiae* for arabinose consumption, resulting in a 3.5-fold increase in specific ethanol productivity. Although the manipulation of *S*. *cerevisiae* strains for higher ethanol production is highly remarked by researchers, a few studies have been conducted regarding the molecular modulations during enhanced and repressed ethanol fermentation. Such research, on the other hand, is critical for improving the efficiency of industrial applications. De Souza et al. [9] reported the presence of stress-response and energy-related genes under optimized fermentation condition supplemented by Mg^2+^ [9]. Exploring for genes that are linked to ethanol tolerance in *Saccharomyces*. *C via* transcriptomic analysis revealed that glucose metabolism and energy related genes are induced by ethanol stress. Moreover, some processes such as oxidative phosphorylation and cellular respiration shown to be affected by ethanol stress [10]. Identifying the molecular basis and dynamics of gene expression profiles related to yeast response in improved bioethanol production conditions is critical for developing new manipulated strains with increased ethanol yield. It also shed light on the mechanisms that yeast uses to improve production. Metal supplements significantly affect the ethanol production associated metabolic pathways in yeast. Among these, zinc, magnesium, manganese, and copper have been extensively studied and shown to have regulatory effects on ethanol production [11–13]. Mg^2+^ ion is involved in phosphorylation, DNA and protein synthesis, cell membrane rigidity and proliferation, along with the potential to increase ethanol accumulation through fermentation [9, 14]. Furthermore, Mg^2+^ may improve the *S*. *cerevisiae* tolerance to high ethanol concentration during glucose and xylose fermentation [15, 16]. *S*. *cerevisiae* medium supplementation with Mg^2+^resulted in a 29% increase in ethanol production through regulating cell wall and membrane related genes expression [16]. *Copper ion* (Cu^2+^) is also known as a critical element for yeast biological functions and involves in a variety of metabolic pathways. For instance, cytochrome c oxidase, a component of oxidative phosphorylation, and superoxide dismutase as an important enzyme contribute to stress regulation, are dependent on Cu^2+^ [17]. Copper stress, on the other hand, caused by an excess of copper, can result in reactive oxygen species (ROS) generation and DNA damage. At high concentrations, it also has a negative impact on cell membrane stability and enzyme activity. [18]. A high copper concentration (1.5 mM) inhibited cell growth, glucose and fructose consumption during fermentation by *S*. *cerevisiae* [19]. However, few studies have been conducted to investigate the effect of Cu^2+^ on the molecular physiology and fermentation ability of *S*. *cerevisiae* cell. Despite their lack of research, Mg and Cu have the potential to modulate the gene expression network involved in the fermentation process.

It would be possible to identify the critical genes and clarify the molecular mechanisms involved in the ethanol production process using bioinformatics-based analysis of the *S*. *cerevisiae* expression dataset. Computational approaches for identifying key genes involved in the fermentation process could elucidate the transcriptomic dynamics of yeast ethanol fermentation and reveal expression signatures contribute to improved production. RapidMiner is one of the most useful and comprehensive mining tools in data science [20]. Different gene selection algorithms, such as Information Gain, Information Gain Ratio, rule induction, support vector machine (SVM), and PCA, are widely used in gene expression analysis using RapidMiner. Machine learning algorithms, both supervised and unsupervised models, are widely used in gene expression data analysis and gene identification [21, 22]. Cheng et al [23] used RapidMiner to preform four machine learning weighting models on gene expression datasets related to Huntington’s disease, including decision tree, rule induction, random forest, and generalized linear algorithms, in order to identify contributing genes to this disorder. Huang et al. [24] conducted Modelling with network features and machine learning approach to identify the functional features that affect the *S*. *cerevisiae* longevity. Mitochondria and cell cycle were found to have determinant role in yeast life span. However, to the best of the authors’ knowledge, no machine learning tools via RapidMiner have been utilized to investigate the molecular basis of metabolic pathways in *S*. *cerevisiae*. Valuable publicly available data related to *S*. *cerevisiae* genome-wide expression experiments could be used for functional genomic analysis through machine learning. Machine learning algorithms’ discriminative ability aids in revealing the unravelling biological process from microarray data sets [20]. In light of the availability of such useful primary data sets and the potential of RapidMiner as an efficient tool for biological data analysis, we used available microarray expression dataset related to *S*. *cerevisiae* supplemented with Copper and Magnesium metal components under fermentation to investigate the underlying molecular basis of fermentation used by *S*. *cerevisiae*. The aim of this study was to identify the critical genes discriminate the improved (Mg^2+^ treatment) and low ethanol production (Cu^2+^ treatment at toxic concentration) and elucidate the transcriptomic response of *S*. *cerevisiae* under these two conditions. *S*. *cerevisiae* transcriptome analysis using data mining and machine learning by both supervised and unsupervised models was used in this study as a novel approach to identify the underlying gene regulation mechanisms that can be used to optimize fermentation performance.

## Materials and methods

### Data collection

For this study available microarray datasets related to yeast fermentation performance under Mg^2+^ (500 mg/L) or Cu^2+^ (1 mg/L) supplementation was used. Microarray data of the industrial yeast *S*. *cerevisiae* JP1 strain downloaded from the GEO repository of the NCBI database (GEO number: GSE75803) was used. To meet the research objective, the probe sets with significant differential expression (concomitant Adj. p < 0.05 and B ≥ 3) were chosen for this study.

### Data cleaning

We used RapidMiner software (RapidMiner Studio 7.6) [25] to enter the 6300-differential expressed probe sets as numerical features, as well as high and low bioethanol as class features. For better processing, inefficient or redundant probe sets with less than or equal to a given standard deviation (SD) threshold (0.1), as well as correlated probe sets (correlation ≥ 0.95), were carefully removed from the dataset. The resulting list, which only contained efficient probe sets, was designated as the Final Cleaned (FCdb) database.

### Attribute weighting algorithms

Eleven attribute weighting algorithms with cut-off ≥ 0.7 were used in the FCdb to identify the most effective probe sets contributing to discriminate ethanol content. Weights close to 1 indicate that a specific probe set in ethanol content is more important. The main probe sets were those determined by the majority of AWAs (intersection of the weighing method). The attribute weighting algorithms used in this investigation, as well as the statistical background description for each one, are as follows (RapidMiner Studio 7.6):

### Weight by information gain and information gain ratio

This algorithm is a well-established superior method for gene selection in microarray data analysis [26, 27]. In this method, the attributes (probe sets) are weighted according to their class label (high or low ethanol production).

### Weight by rule

Based on a single rule and the relationship between attributes (genes) and considering the errors, the weight of each attribute is measured through rule algorithm [28] and is used as a selective method for microarray analysis.

### Weight by deviation, weight by correlation and weight by chi squared statistic

The standard deviation of attributes is used as a weighting parameter in the deviation weighting method. The correlation method, on the other hand, weighs the label attributes based on the correlation. In addition, for labeling the attributes, we used the Chi Squared Statistic weighting algorithm, which takes the Chi squared into account.

### Weight by Gini index and weight by uncertainty

Due to the label attribute in this model, the weight of attributes is determined by measuring the Gini coefficient as an inequality index of sample data. According to each attribute, the lower the Gini index of the attribute, the more equal dispersion among attributes is considered. The weight for uncertainty model, on the other hand, is determined using the symmetrical uncertainty due to the class attribute.

### Weight by relief

This model is one of the most reliable algorithms for weighting genes because it is based on the determination of values between probe sets of the same and different classes in a short distance.

### Weight by SVM

SVM is one of the most powerful classification models for gene expression analysis [21]. The SVM method weighs attributes using the coefficients of the normal vector of a linear SVM.

### Weight by PCA

This model performs attribute weighting due to the class attribute based on the component number parameter of PCA and the value of the components.

### Decision tree models

Eleven new datasets were generated using the entire probe sets with weight >0.70. They were annotated based on the models used for attribute weighting (Relief, Information gain, Uncertainty, Information gain ratio, Chi Squared, Rule, Correlation, Deviation, SVM and PCA, Gini index). Random Tree, Decision Tree, Decision Stump, and Random Forest were the tree induction models used for 12 datasets (FCdb and 11 datasets produced by specific weighting algorithms). Each model had four criteria (Gini Index, Gain Ratio Information Gain, and Accuracy). We used a ten-fold validation algorithm with appropriate sampling to create trees with RapidMiner. The performance of the model was evaluated and used to compare various models based on the accuracy of each model in identifying the target variable (high and low bioethanol content) and according to the attribute variables (normal expression of the probe set). Performance is expressed as a measure of model accuracy in this case. We calculated the accuracy by dividing the number of correct predictions by the total number of samples. The value of the attribute accuracy that is expected to be the same as the value of the labeled attribute is referred to as the correct prediction. These models were used with a minimum gain of 0.1 to obtain a split and a maximum tree depth of 20. For pruning 0.25 confidence level was considered with a pessimistic error calculation.

### Unsupervised analysis of the top ranked probe sets derived by supervised AWAs

Unsupervised principal component analysis (PCA) and hierarchical clustering heatmap were used to evaluate the power of top-ranked probe sets which differentiate the fermentation under different supplementation treatments. For unsupervised analysis, a web-based tool Clustvis (https://biit.cs.ut.ee/clustvis/) was used [29]. The PCA analysis was carried out in the PCA Methods R package using unit variance scaling on rows and Singular Value Decomposition (SVD) with the imputation method. The clustering heatmap was created with the pheatmap R package (version 0.7.7). The clustering heatmap was constructed using correlation, Pearson correlation subtracted from 1, and the average distance of all possible pairs [29].

### Kyoto Encyclopedia of Genes and Genomes (KEGG) pathway enrichment analysis

The pathway enrichment analysis was carried out using YeastEnrichr (https://maayanlab.cloud/YeastEnrichr/) [30, 31]. The biochemical pathways related to key probe sets were identified using the KEGG2019 database. Pathways with p-value < 0.1 were considered significant.

### Exploring for transcription factors among top-ranked genes and regulator cluster analysis

We used yeastract database (http://www.yeastract.com/formrankbyhomotf.php) to identify transcription factors (TFs) among the 171 probe sets identified by at least 5 attribute weighting algorithms [32]. The TFs and their target genes were identified using this tool based on DNA binding sites and expression evidence. Furthermore, we used the regulator DB database (http://wyrickbioinfo2.smb.wsu.edu/cgi-bin/RegulatorDB/cgi/home.pl) to run regulator cluster diagram to determine the regulatory effect of the identified TFs on the target genes [33, 34]. It provides data on mutant regulator expression for selected regulators and target genes.

### Independent validation of top-ranked genes using different microarray expression datasets

For independent validation of the top-ranked genes determined by supervised attribute weighting models, impartial samples of high or low ethanol production from microarray experiment with GEO accession of GSE78759 and GSE5185 [35, 36] were selected. The original experiments regarding GSE78759 had been designed to investigate the transcriptomic changes occurred in two ethanol-tolerant strains with maximum ethanol production rate, selected through evolutionary engineering, in comparison with their reference as low ethanol producing strains. The other dataset, GSE5185, was related to improved Ethanol producing mutant generated by transcription machinery engineering compared with the wild strain as lower ethanol producing. We used 5 samples including GSM2075761, GSM2075762, GSM2075763, GSM2075764, GSM2075765 as high ethanol producing strains and 3 samples including GSM2075758, GSM2075759, and GSM2075760 as low ethanol producing from the first dataset as well as GSM116819 and GSM116820 as high ethanol producing and GSM116815, GSM116816, GSM116817 as low ethanol samples from the second datasets. The 171 probe sets which were identified by AWAs were selected among the samples and the unsupervised principal component analysis (PCA) were used to evaluate the power of top-ranked probe sets to separate the low and high samples. Besides, validation of the top-ranked genes was implemented using a leave-one-out cross-validation (LOOCV) on expression values of the top-ranked genes. Attribute (expression values of 171 probe sets) were chosen using the LOOCV mode in which expression matrix of one sample discarded for the test and the remaining parts for training. Discrimination was performed based on Logistic function to model a binary classification of samples according to their gene expression values.

## Results

### Ranking probe sets by AWAs

After cleaning 6300 probe sets by RapidMiner, we obtained 1813 probe sets. Eleven AWAs were used to identify informative probe sets. Following AWAs analysis, 171 probe sets were identified by at least 5 attribute weighting algorithms (S1A File). Furthermore, there were distinct probe sets classified by at least five algorithms that respond discriminatively to supplement treatment and/or are particularly related to ethanol production during fermentation. Sheet B of the S1 File contains the probe sets as well as the AWAs used to identify the probe sets. Some of the informative probe sets were recognized to be involved in carbohydrate metabolism, TCA cycle, oxidative phosphorylation, and ethanol fermentation while others were related to stress responses, cell membrane structure, and cell growth which could be indirectly effective in ethanol production. Some of the top informative genes are presented in Table 1. However, it should be noted that some of discriminative probe sets which respond to Mg^2+^ and Cu^2+^ supplementation may not be required in or influence the ethanol production process.

**Table 1:** Some of the informative probe sets identified by at least four AWAs.

probe sets	Standard Gene Name	AWAs Names	AWAs number	Gene Name
A_06_P4554	MRP8	Chi Square Statistic, Correlation, Gini Index, Information Gain, Information Gain ratio, PCA, Relief, Rule, SVM, Uncertainty	10	Uncharacterized, response to stress
A_06_P1016	OLI1	Chi Square Statistic, Correlation, Gini Index, Information Gain, Information Gain ratio, PCA, Relief, Rule, SVM, Uncertainty	10	ATP synthase subunit 9, mitochondrial;OLI1;ortholog
A_06_P1397	ADH5	Chi Square Statistic, Correlation, Gini Index, Information Gain, Information Gain ratio, PCA, Rule, SVM, Uncertainty	9	Alcohol dehydrogenase 5;ADH5;ortholog
A_06_P1238	PKC1	Chi Square Statistic, Correlation, Gini Index, Information Gain, Information Gain ratio, PCA, Rule, SVM	8	Protein serine/threonine kinase
A_06_P3384	GTR2	Chi Square Statistic, Correlation, Gini Index, Information Gain, Information Gain ratio, PCA, Rule, SVM, Uncertainty	9	GTP-binding protein
A_06_P1063	CYC3	Chi Square Statistic, Correlation, Gini Index, Information Gain, Information Gain ratio, Relief, Rule, SVM, Uncertainty	9	Cytochrome c heme lyase
A_06_P1003	COX1	Chi Square Statistic, Correlation, Gini Index, Information Gain, Information Gain ratio, Rule, SVM, Uncertainty	8	cytochrome c oxidase
A_06_P2810	PDA1	Chi Square Statistic, Correlation, Gini Index, Information Gain, Information Gain ratio, Rule, SVM	7	Pyruvate dehydrogenase E1 component subunit alpha, mitochondrial;PDA1;ortholog
A_06_P2931	QCR6	Chi Square Statistic, Correlation, PCA, Rule, SVM	5	Cytochrome b-c1 complex subunit 6;QCR6;ortholog
A_06_P6820	ALD6	Chi Square Statistic, PCA, Rule, Uncertainty	4	Magnesium-activated aldehyde dehydrogenase, cytosolic;ALD6;ortholog

### Decision tree models

Sixteen different decision tree models were applied on twelve datasets (eleven new datasets produced by trimming FCdb based on a weight ≥ 0.7 given by each attribute weighting approaches, as well as FCdb) were used to achieve pattern recognition between important genes as well as with the genes with the highest distinguishing power. The performance of each model was evaluated and the lowest and highest performances were 0% and 100%, respectively (S1C and S1D File). The entire model trees were characterized with root node, branches and leaf nodes (class label; high or low ethanol). The topmost node in the tree is the root node. Genes in the root of decision tree have stronger effects on determining the general data pattern than genes in the tree branches. There were 17 probe sets with 100% performance in the roots of decision tree models Table 2. The root list contains *OLI1*, *CYS3*, *HTB2*, *CGR1*, *CYC3*, *RPS13*, *OST4* and *COX1* genes with known biological process. Albeit probably contributed to ethanol fermentation, the biological processes of the other genes determined as the roots of decision trees are not known yet.

**Table 2:** Decision tree models roots identified as exhibited 100% performance.

elements	GENE_SYMBOL	AWS	DESCRIPTION
A_06_P1016	OLI1	10	BioProcess = ATP synthesis coupled proton transport
A_06_P2475	CWC21	9	BioProcess = biological_process unknown
A_06_P1002	ORF:Q0017	9	BioProcess = biological_process unknown
A_06_P1034	CYS3	9	BioProcess = sulfur amino acid metabolism*
A_06_P1131	HTB2	8	BioProcess = chromatin assembly/disassembly
A_06_P1068	KRE23	9	BioProcess = biological_process unknown
A_06_P2984	CGR1	9	BioProcess = rRNA processing*
A_06_P1298	ORF:YBR051W	9	BioProcess = biological_process unknown
A_06_P1524	ORF:YBR270C	8	BioProcess = biological_process unknown
A_06_P3287	ORF:YGR067C	9	BioProcess = biological_process unknown
A_06_P1063	CYC3	9	BioProcess = not yet annotated
A_06_P2051	RPS13	9	BioProcess = protein biosynthesis
A_06_P1967	OST4	10	BioProcess = not yet annotated
A_06_P1049	ORF:YAL027W	9	BioProcess = biological_process unknown
A_06_P1003	COX1	8	BioProcess = aerobic respiration
A_06_P2023	ORF:YDR036C	7	BioProcess = biological_process unknown
A_06_P1023	ORF:Q0297	8	BioProcess = biological_process unknown

### Unsupervised analysis

**Fig 1:**
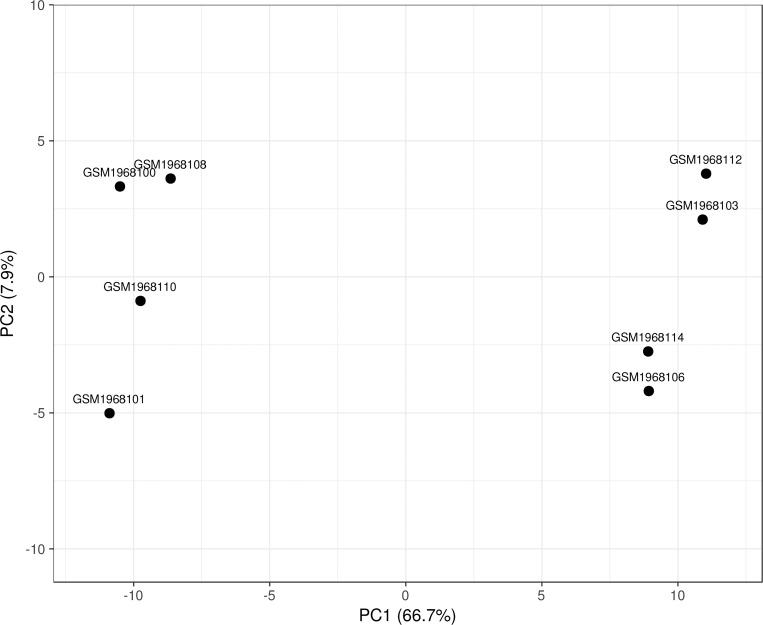
Two-dimensional plot related to the first two principal components. GSM1968101, GSM1968110, GSM1968100 and GSM1968108 are samples related to Mg^2+^ supplementation. GSM1968106, GSM1968114, GSM1968103 and GSM1968112 are samples related to Cu^2+^ supplementation.

**Fig 2:**
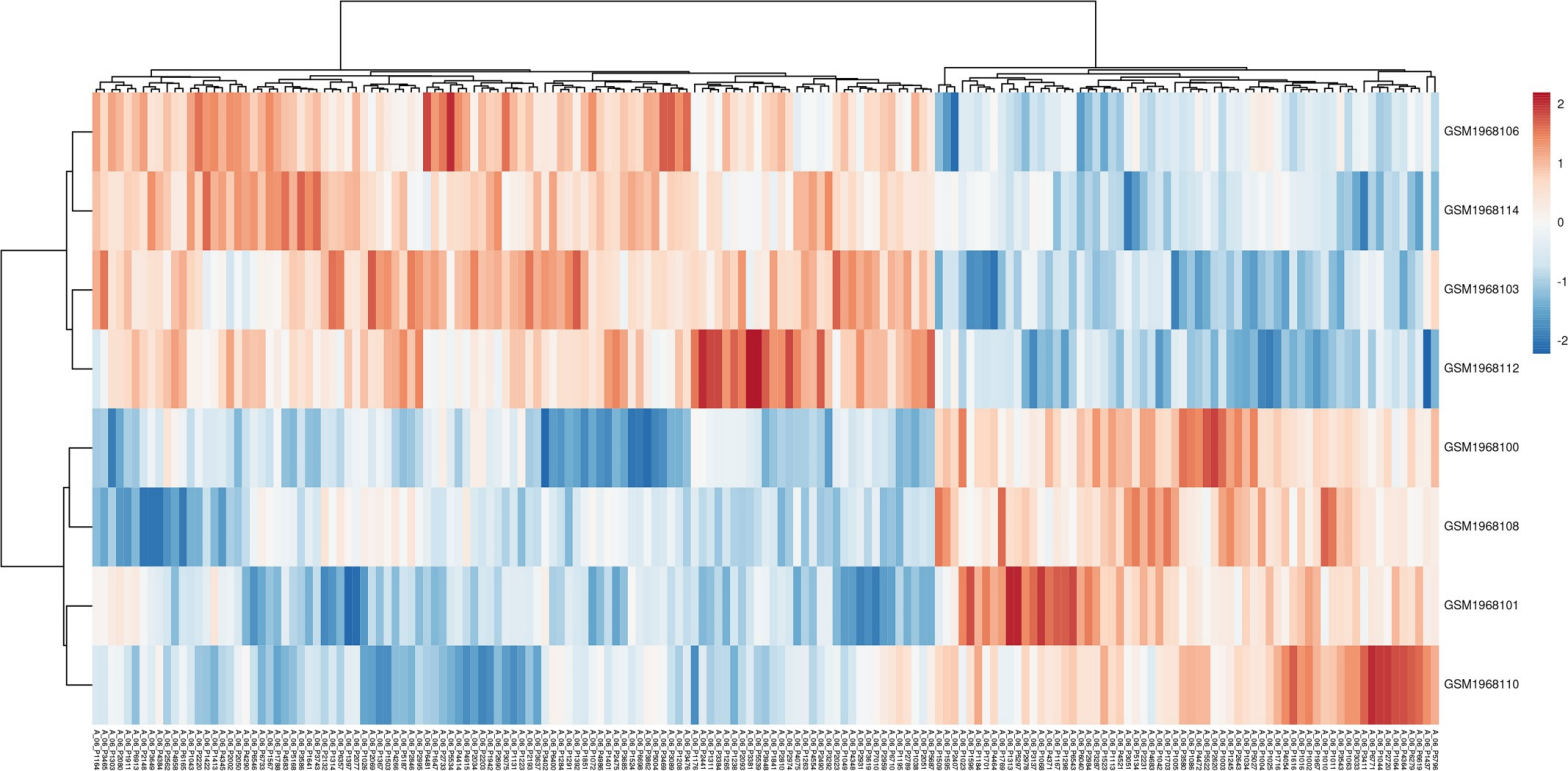
The heatmap related to 171 probe sets which were recognized by at least 5 attribute weighting algorithms (AWAs). Each row corresponds to the different samples including Mg^2+^ (high ethanol production) and Cu^2+^ supplementation (repressed ethanol production). Columns exhibits hierarchically clustered probe sets. The normalized intensity expressions of probe sets were shown as a color scale. The up and down-expression levels were represented as red and blue scales, respectively.

As a complementary confirmation, the 171 top ranked probe sets were validated using PCA and hierarchical clustering heatmap, identified with supervised attribute weighting models. According to the results, the 171 significant probe sets could accurately differentiate between two different fermentation conditions, thus confirming the significance and accuracy of the identified probe sets (Fig 1). In particular, the captured variances with the first two components on all recognized 6031 probe sets and informative 171 probe sets were up to 75% and 50%, respectively. Furthermore, it could efficiently separate informative 171 probe sets under Cu^2+^ or Mg^2+^ supplementation in the hierarchical clustering heat map, (Fig 1). In total, 64 probe sets were up and down regulated by Mg^2+^ and Cu^2+^, while 108 probe sets were up and down regulated through Cu and Mg supplementation, respectively (Fig 2).

### Pathway enrichment analysis of genes

A total number of 21significant terms were recognized and enriched in ranked probe sets (adjusted p-value <0.1). Some probe sets including *PDA1* and *CYC3* participated in multiple pathways. Significant related enriched pathways such as Porphyrin metabolism, Oxidative phosphorylation, Glycolysis, Amino sugar and nucleotide sugar metabolism, Cell cycle, MAPK signaling pathway and Citrate cycle (TCA cycle) were identified using the KEGG enrichment analysis. The porphyrin metabolism pathway shown to be the most significant defined by probe sets list. Confirmed by Qi et al. [37], the porphyrin biosynthesis is reported to be associated with ethanol fermentation in yeast through controlling ROS level under stress condition. On the other hand, the topmost genes, including *COX1* and *OLI1*, that were ranked by the highest number of AWAs involved in Oxidative phosphorylation pathway. This suggest that this pathway is crucially regulated by *S*. *cerevisiae* Cu^2+^ and Mg^2+^ supplementation experiment. The enriched pathways and the related genes are presented in Table 3.

**Table 3:** KEGG enrichment analysis of 171 probe sets. The significant pathways with adjusted p-value < 0.1 are represented.

Term	Adjusted p-value	Genes
Porphyrin and chlorophyll metabolism	0.000582985	HEM2;HEM12;CYC3;YFH1
Oxidative phosphorylation	0.007415084	OLI1;QCR6;ATP6;COX1;ATP2
Endocytosis	0.007415084	CAP1;APL3;LAS17;ARC15;VPS25
RNA degradation	0.025376563	POP2;RRP42;SSQ1;CCR4
Meiosis	0.049279656	CLN3;HMRA2;MSN4;APC9;TPD3
Autophagy	0.049279656	KCS1;VPS8;MSN4;PEP4
Ubiquitin mediated proteolysis	0.049279656	UBC13;UBC6;APC9
Protein processing in endoplasmic reticulum	0.049279656	OST4;UBC6;PDI1;SSE2
Glycolysis / Gluconeogenesis	0.064176371	PDA1;PGM2;ADH5
Galactose metabolism	0.072088565	GAL7;PGM2
Phosphatidylinositol signaling system	0.072088565	KCS1;PKC1
Amino sugar and nucleotide sugar metabolism	0.075323668	GAL7;PGM2
Spliceosome	0.075323668	PRP43;ECM2;PRP8
MAPK signaling pathway	0.072088565	TUP1;MKC7;MSN4;PKC1
Pentose phosphate pathway	0.075323668	SOL4;PGM2
Alanine, aspartate and glutamate metabolism	0.075323668	GDH3;NIT3
Cell cycle	0.075323668	CLN3;TUP1;APC9;TPD3
Citrate cycle (TCA cycle)	0.075323668	PDA1;LSC2
Ribosome biogenesis in eukaryotes	0.075323668	UTP15;CKB1;RIO1
Glycine, serine and threonine metabolism	0.075323668	SER1;CYS3

### Identification of transcription factors and their targets

**Fig 3:**
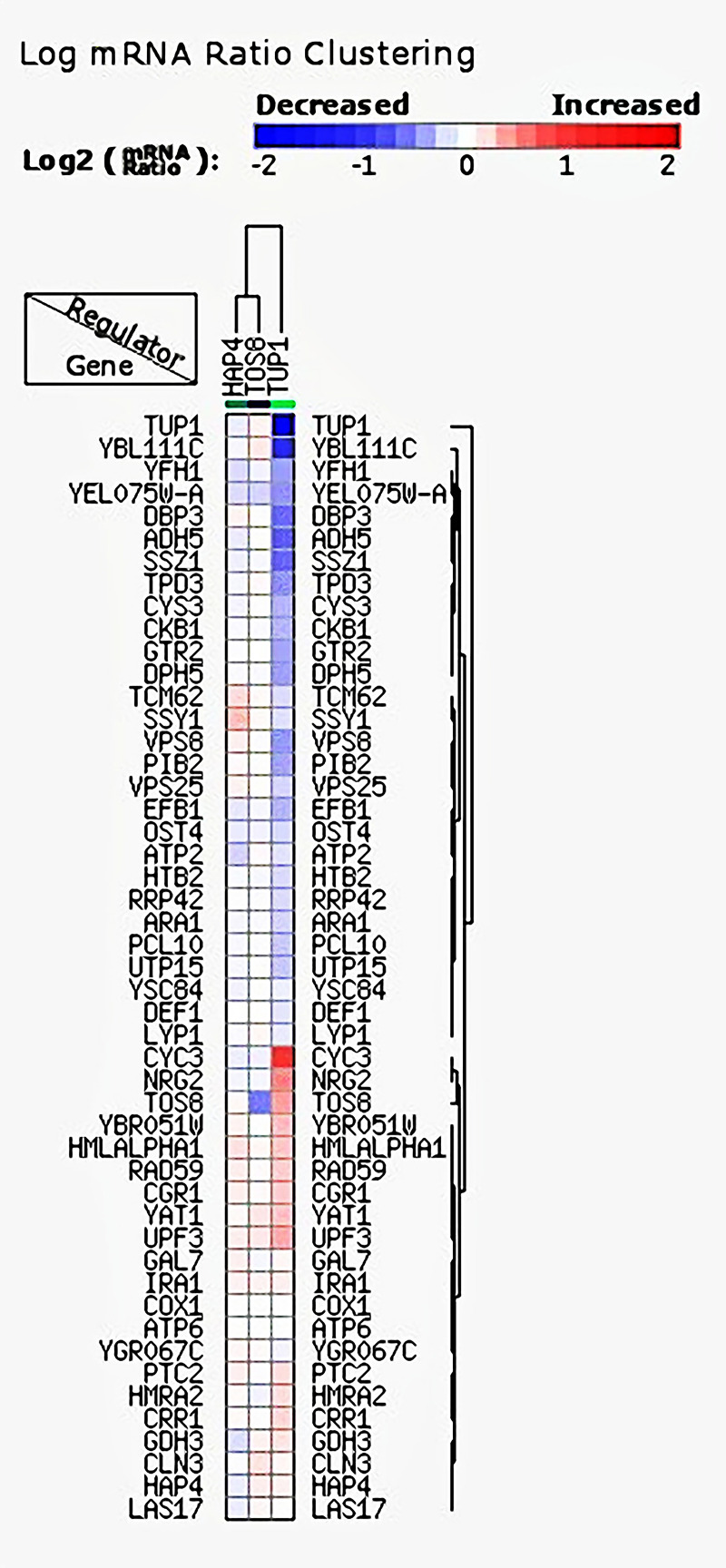
The regulatory clustering heatmap related to genes targeted by identified transcription factors *Hap4p* and *Tup1* and *TOS8*. The cluster is represented as the log mRNA ratio of each target gene in each regulator mutant.

Among the 171 informative probe sets identified by yeastract analysis were seven transcription factors: *YGR067C*, *HAP4*, *NRG2*, *TUP1*, *TOS8*, *MSN4*, *and PDC2*. Surprisingly, the targets of the identified transcription factors were discovered among the 171 genes identified by RapidMiner analysis and ranked by at least 5 algorithms (S1E File). These findings support the AWAs’ ability to correctly identify top-ranked probe sets. Furthermore, regulator clustering related to TFs and their targets (both ranked by at least eight algorithms) was performed to demonstrate the effect of top-ranked TFs on top-ranked target genes based on the transcription factors mutants. The results showed that *Hap4p*, *Tup1*, and *TOS8* mutants resulted in different ratios of up and down-regulation of target genes (Fig 3). Although *Hap4* and *TOS8* resulted in down or up regulation of target genes, their effect on none of target genes was significant. According to the findings, the *Tup1* transcription factor has the greatest impact on the target genes expression. The *Tup1* knocked out mutant significantly induce the expression of *CYC3 (YAL039C)*, while causing highest level of down regulation of *YBL111C*.

### Validation of top-ranked genes through different *S*. *cerevisiae* strains datasets

The top-ranked genes derived by supervised attribute weighting models showed high reproducibility in an independent microarray experiment related to evolved ethanol-tolerant clones derived from *S*. *cerevisiae* strains CEN.PK with high ethanol production rate, in comparison with the reference strain. Similar results were obtained from the evaluation of informative identified genes based on GSE5185 dataset which belongs to higher ethanol producing mutant strain *spt15* in comparison with its reference. Noticeably, the captured variance with the first two components on 171 probe sets was as high as 78.60% and 70.40% in the GSE5185 and GSE78759 datasets, reinforcing that the identified top-ranked genes were able to accurately discriminate between low ethanol samples and high ethanol ones (S1 Fig). Also, according to LOOCV analysis result, the top-ranked genes were able to identify low and high ethanol samples with classification accuracy of 100%. The classification performance was further evaluated based on the following parameters: Precision = 1; Recall = 1; F-Measure = 1; and ROC Area = 1 (S1G File).

## Discussion

In this study, machine learning and decision tree models were used to analyze the transcriptome of *S*. *cerevisiae* during the fermentation process in two conditions: repressed ethanol production and high ethanol production supplemented with Cu^2+^ and Mg^2+^ respectively. Indeed, for the most accurate prediction methods, we used both supervised and unsupervised models. In summary, we used 11 supervised models to achieve high accuracy results. In addition, a PCA analysis as an unsupervised model and a hierarchical clustering heat map were used to validate the 171 top-ranked probe sets identified by supervised-based models. In parallel, to validate the reliability of identified probe sets in another *S*. *cerevisiae* strain, we used to evaluate the reproducibility of the results by exploring the discriminative potential of them via PCA and LOOCV analysis based on different gene expression datasets related to another *S*. *cerevisiae* strain and different experiment. LOOCV and unsupervised analysis of the data using PCA demonstrated the potential of 171 probe sets to discriminate between the low and high ethanol samples from independent microarray experiments. The captured variance with the first two components by PCA analysis on 171 probe sets was high (78.60% and 70.40%). These findings imply that AWAs applied to FCdb can substantially reduce the dimensionality of the data by eliminating probe sets that are unlikely to be linked to ethanol production, leading to identification of the most reliable probe sets associated with ethanol production.

**Fig 4:**
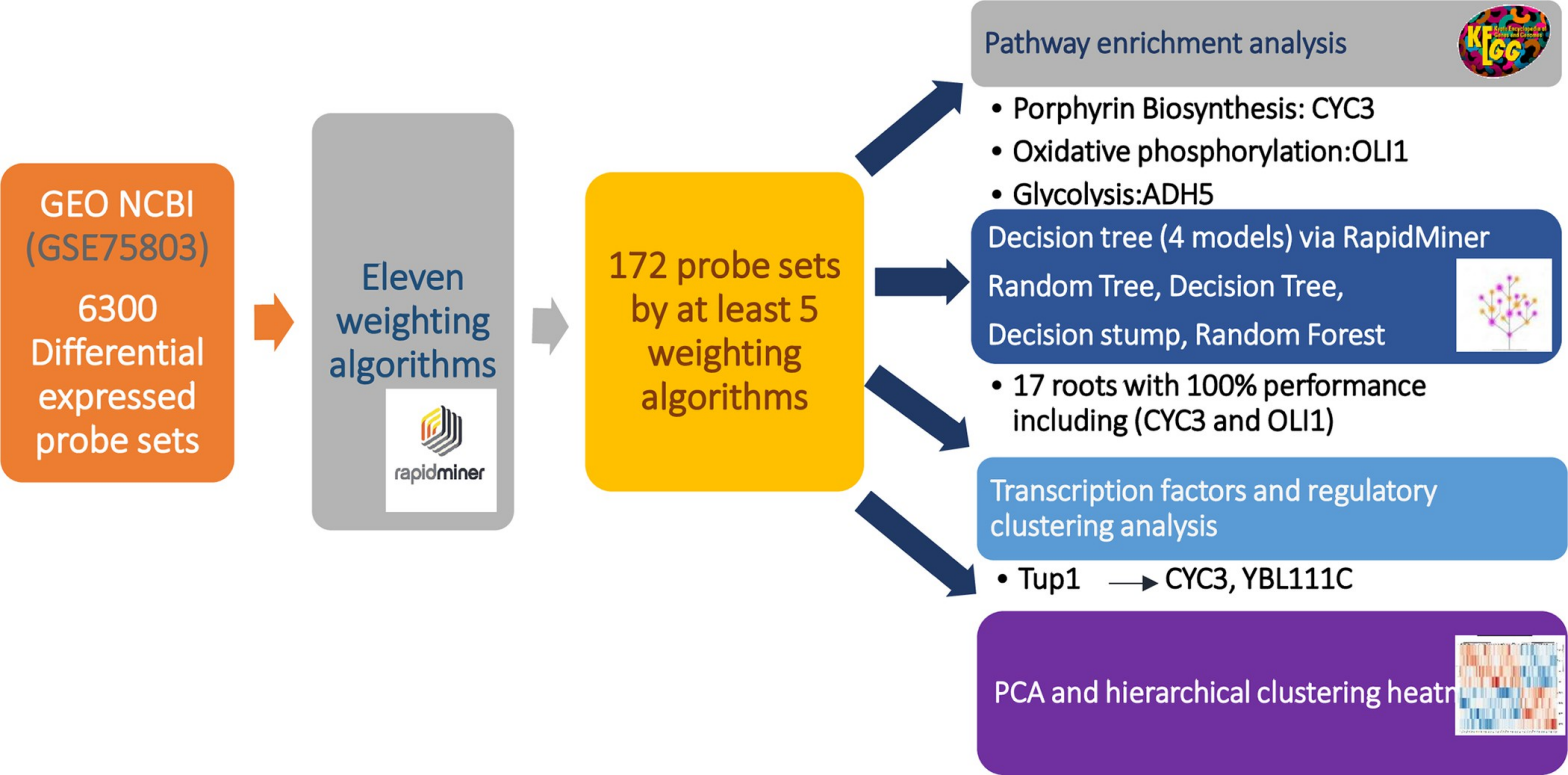
The schematic illustrates the methodology of the study with summarized results.

Furthermore, we used pathway enrichment, transcription factor and regulatory analysis to validate the machine learning analysis results (Fig 4). According to RapidMiner-assisted analysis, some probe sets were identified as playing a distinct role in ethanol production. Nonetheless, it should be noted that the function of some identified probe sets has not yet been clarified, despite the fact that they may be critical in ethanol production. *ADH5* or Alcohol dehydrogenase, which was weighted by 9 algorithms and classified in Glycolysis / Gluconeogenesis by KEGG enrichment analysis, contributes to ethanol production by reducing acetaldehyde to ethanol [38]. *OLI1* was distinguished by ten algorithms and enriched by Oxidative phosphorylation term, which encodes F0-ATP synthase subunit c and generates ATP in yeast mitochondria [39]. Cu^2+^ in toxic concentration, is known to have a negative effect on mitochondrial respiratory components, as it repressed the respiratory chain in *PC12* and liver cells at toxic doses [40, 41]. That is most likely the main reason for the down regulation of *OLI1*, which is an important component of the oxidative phosphorylation pathway when exposed to Cu^2+^ treatment. RNA-seq analysis revealed that this gene was enriched as a significant gene between the wild and high glucose tolerant mutant strains of *S*. *cerevisiae* [42]. In addition to this gene, *COX1* has an AWA weight of 8 and is involved in the final electron chain reaction in the respiratory system [43]. It encodes one of the cytochrome c oxidase subunits and, like *OLI1*, has been shown to be repressed by Cu^2+^ treatment. *PDAI* encodes alpha subunit of pyruvate dehydrogenase and converts the pyruvate to acetyl-CoA through oxidative decarboxylation [44]. This gene was found to be enriched by the Glycolysis/Gluconeogenesis pathway by seven weighting algorithms used in this study. *PDAI* directs the pyruvate metabolism to Acetyl- COA in mitochondria to provide the TCA cycle substrate. In other words, directing the pyruvate to TCA cycle *PDAI* keeps pyruvate from being consumed in the fermentation process or ethanol production. *PDAI* was down regulated in Mg-containing medium, which accounts for improved ethanol production, and was upregulated in the repressed fermentation condition, by Cu. *QCR6* is a subunit of cytochrome bc1 complex and contributes to oxidative phosphorylation. Cytochrome C is known to be activated by Cu metal ion [45]. *QCR6* was up regulated, as expected, by Cu supplementation. Similarly, in Pichia stipites, cytochrome bc1 disruption resulted in increased ethanol production. [46]. Granados-Arvizu et al [47] also concluded that cytochrome bc_1_ complex repression would be a promising way to enhance ethanol production in *Saccharomyces stipites*. *ALD6* or Aldehyde dehydrogenases is activated by Mg and have a distinct role in the formation of acetate from pyruvate in an alternate pyruvate dehydrogenase bypass pathway [48]. Here, *ALD6* expression was found to be increased with Mg supplementation, which corresponded to the activation of this enzyme by Mg^2+^. Since it consumes the acetaldehyde source that ADH enzymes can use to produce ethanol, deleting *ALD6* via clustered regularly interspaced short palindromic repeats (CRISPR/Cas9) genome editing resulted in increased ethanol production [49].

Based on decision tree analysis, 17 identified roots exhibited 100% performance, some of which have unknown molecular functions and have yet to be characterized. Surprisingly, *OLI1* and *CYC3* were identified by the highest attribute weighting algorithms (10 and 9), were enriched by the second most significant biochemical pathway, and were also identified as decision tree model roots with 100% performance. *COX1* is also shown as a complete root, but it is identified using 8 weighting algorithms. As previously stated, *OLI1* is an F0-ATP synthase subunit c that contributes to the electron transport chain. *CYC3* is also known as Cytochrome c heme lyase and has a strong sensitivity to ethanol. Indeed, the null mutants for this gene showed ethanol sensitivity. Both of these genes are involved in ATP generation and are up regulated in Mg supplemented medium according to the results. Nonetheless, it has been established that Mg^2+^ has an effect on energy metabolism and ATP production in the cell [50].

The Kegg enrichment analysis demonstrated that the topmost significant pathways, porphyrin biosynthesis and oxidative phosphorylation, were enriched in *CYC3*, *OLI1* and *COX1*. Interestingly, these genes were also identified based on AWAs and decision tree models analysis. Besides, Cell cycle and division, as well as Ribosome biogenesis, are identified as significant terms in the KEGG pathway enrichment. They may have an impact on ethanol production even though they do not directly contribute to the fermentation bioprocess. For example, in addition to its role in yeast cell growth and proliferation, which affects ethanol production, ribosome biogenesis is predicted to be associated with fermentation, and some related genes, such as *SFP1* are thought to be involved in glycolysis control as well [38, 51]. Nonetheless, significant phosphatidylinositol signaling and mitogen-activated protein kinase (MAPK) signaling pathways identified in this study by enrichment analysis were reported to be responsible for cell proliferation/growth regulation and critical for stress responses [52, 53]. *PKC1* which was attributed by 8 algorithms and remarkably enriched in phosphatidylinositol signaling system is a serine/threonine kinase which is suggested to have role in response to copper toxicity since it was upregulated in Cu^2+^ supplementation or reduced ethanol production according to heatmap clustering. Confirming this finding, Zhou et al. [52] reported that 5-hydroxymethyl-2-furaldehyde, which is toxic to industrial fermentative *S*. *cerevisiae* strain, increases the expression of *PKC1* gene. Furthermore, according to AWAs analysis, some genes are involved in stress responses, cell growth and proliferation, protein synthesis, fatty acids and lipid metabolism, all of which may contribute to ethanol production efficiency. *MRP8* was assigned by ten algorithms as a response to cell wall stress, and its expression has been reported to be induced under stress conditions [54]. Its function, however, is unknown. Here, Cu^2+^ supplementation also induces the expression of this gene in response to the stress condition caused by copper. *GTR2*, a GTPase subunit, was weighted using nine algorithms. It is suggested in this study that it contributes to tolerance response to Cu supplementation because it was up regulated by copper. As an implication for this result, the null mutant related to *GTR* gene showed decreased resistance to Zn metal at inhibitory amount [55].

According to the crucial role of TFs in gene expression regulation and to confirm the results obtained from attribute weighting algorithms analysis, the TFs and their targets were explored among 171 probe sets. According to the regulatory clustering analysis, *Tup1* has a significant effect on the top-ranked target genes. *Tup1* is a transcriptional repressor in *S*. *cerevisiae* has the ability to repress target genes via various molecular mechanisms, and it contributes to carbon catabolite repression of transcription by glucose [56, 57]. Regarding the results of this study on regulatory clustering analysis, the *Tup1* mutant caused decreased expression in some of the target genes and up regulation in others. In details, according to the regulatory analysis the *Tup1* deletion most significantly resulted in the downregulation of *YBL111C* whose biological function is not known. Although the *Tup1* deletion affect the ADH5, the expression change was not significant. On the other hand, the *TUP1* knock out resulted in significant upregulation of *YAL039C* (*CYC3*). Indeed, the *CYC3* gene, which was confirmed by the greatest number of AWAs and a decision tree model, was also shown to be a top target of the transcription factor involved in ethanol production responses in this study. *HAP4* is a transcription factor involved in the regulation of the respiratory genes’ expression and ethanol tolerance. The role of *TUP1* and *HAP4* in glucose fermentation have been studied and recently confirmed in thermos-tolerant yeast, *Ogataea polymorpha* [57]. Moreover, the overexpression of *HAP4* gene caused enhanced glucose consumption and ethanol production in *S*. *cerevisiae* [58, 59]. In this study, the *HAP4* gene was also identified as top-ranked gene attributed by nine AWAs. Although the results confirm its involvement in the identified probe sets regulation, it does not demonstrate significant up or down-regulation effect on the target genes. Altogether, *OLI1*, *CYC3*, *COX1 and ADH5* were ranked as the most critical genes in the differentiation of two improved and repressed ethanol production conditions because they were the most frequently identified genes across analyses. These important findings shed light on the complex pathways and regulatory responses that genes use to contribute to ethanol production. However, additional experimental analysis could fully clarify the results. Overall, the findings of this study could be used to further investigate the possibility of improving ethanol through overexpression or knock out strategies. Further experimental investigations, including overexpression and knockout studies as well as gene expression analysis by qRT-PCR are required to confirm the identified genes.

## Conclusion

This investigation provides a significant understanding of the *S*. *cerevisiae* cell molecular response under low and high ethanol production. Preforming eleven machine learning algorithms via RapidMiner on 6300 probe sets related to *S*. *cerevisiae* transcriptomic data under low and high ethanol production, 171 discriminative probe sets were identified. Besides, PCA and hierarchical clustering confirm the accuracy of the supervised discriminating methods. Through different computational analyses including attribute weighting algorithms, decision tree models, unsupervised models, pathway enrichment, and regulatory analysis, prominent genes such as *OLI1*, *CYC3*, *COX1*, *and ADH5* were recognized to be involved in ethanol production level. The results of this study also provide insight into the potential of genes that could be utilized in ethanol production enhancement programs. However, further experimental evaluations are crucial to confirm the findings.

## Supporting information

S1 File A and B. The list of the probe sets along with the attribute weighting algorithms (AWAs) through which the probe sets were identified. C and D. The decision tree models (performance and roots). E. The list of identified transcription factors and their targets. G. Leave-one-out cross-validation (LOOCV) analysis on the top-ranked discriminative genes.
(XLSX)

S1 Fig Two-dimensional plot related to the first two principal components based on 171 identified probe sets. (TIF)
