# Supplementary figures and images for: Mining transcriptomic data to identify Saccharomyces cerevisiae signatures related to improved and repressed ethanol production under fermentation
Source: PLoS One. 2022 Jul 26;17(7):e0259476. doi: 10.1371/journal.pone.0259476 (PMC9321456; doi:10.1371/journal.pone.0259476)

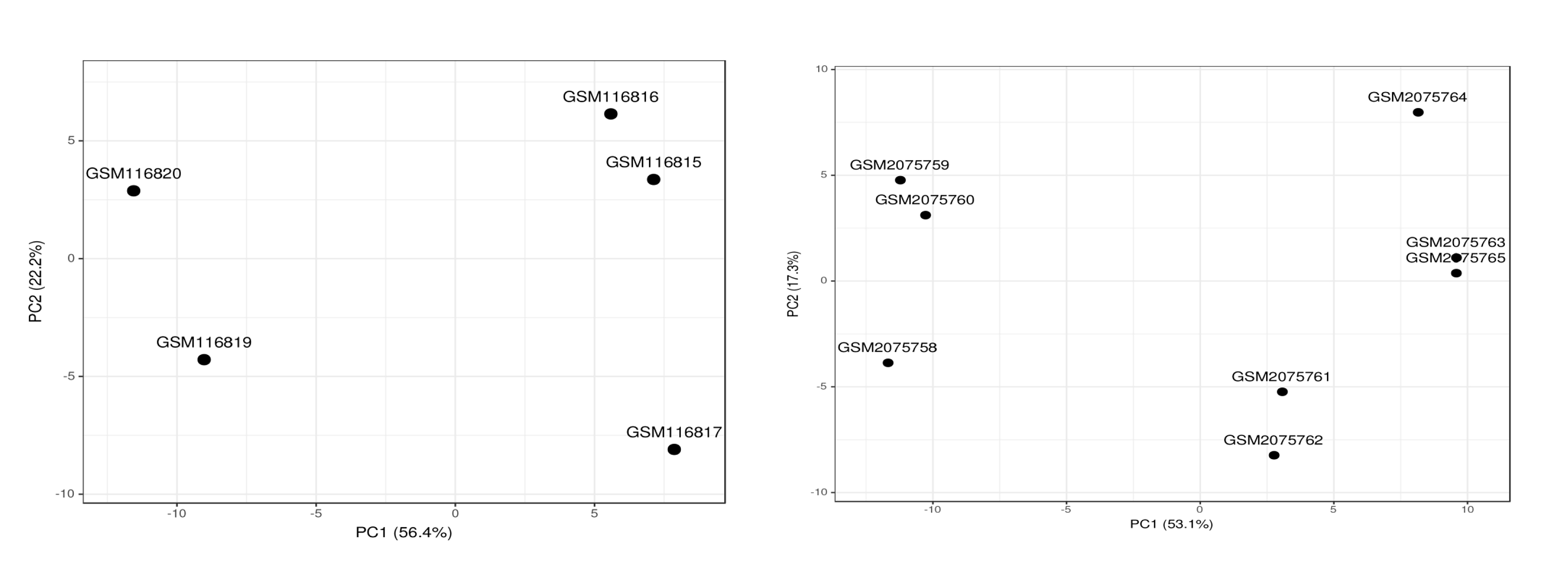

Supplement: S1 Fig — (TIF) [file pone.0259476.s002.tif]
